# Clinical Audit of Rapid Tranquilisation in Mental Health Services for Older People (MHSOP)

**DOI:** 10.1192/bjo.2024.610

**Published:** 2024-08-01

**Authors:** Daniel Hern, Tahir Salim, Mabel Oduma, Wren Langford

**Affiliations:** TEWV, Bishop Auckland, United Kingdom

## Abstract

**Aims:**

Rapid Tranquilisation (RT) is the parenteral (intramuscular) administration of medication to calm or sedate an agitated, violent or aggressive patient in a timely and safe manner. This audit specifically looks at the clinical practice in the use of rapid tranquilisation in inpatient MHSOP against trust policy. The aim of this audit is to assess the effectiveness of RT and if other methods of de-escalation are being utilized first to provide better care for patients and utilize the least restrictive management options possible.

**Methods:**

The audit was registered, and care was taken to uphold ethics, access patient information appropriately, and to ensure that data collected was both relevant and ensured confidentiality. All incidents of RT were identified across both wards of Auckland Park Hospital from the period of August to October 2023. DATIX numbers were identified to show incidents of RT from the specified period, these numbers were used to identify patient ID with liaison with relevant staff members. Patient ID was used to review the incident, specifically to investigate de-escalation techniques documented and effectiveness of RT. Only parenteral RT incidents were included to assess if appropriate measures were taken beforehand.

**Results:**

A total of six Incident reports were identified over the three-month period. In all cases the choice and dose of the medication was within the current recommendations. 33% of incidents utilised promethazine 25mg while the other 66% utilised lorazepam either 1mg or 500mcg. All patients had baseline observations recorded on NEWS chart prior to the incident, however only 33% of incidents involved full recordings of observations at appropriate intervals on the NEWS chart. The reason for this in all cases was due to patient refusing observations which was documented. There were no documented side effects but 33% of incidents involved a raised NEWS score post RT. In all cases the NEWS score resolved spontaneously within the post RT monitoring period. In 100% of incidents de-escalation techniques were utilised and documented and evidence of post RT debrief with the patient was shown. 66% of incidents involved a medication review post RT as per recommendations.

**Conclusion:**

Guidelines are being followed with good effect regarding RT in MHSOP. It is important to always undertake nonpharmacological de-escalation methods prior to considering RT which is reflected in the low numbers of RT during this period. Recommendations are made to follow local guidance as well as to exhaust nonpharmacological de-escalation methods to reduce the need for RT.

